# Modulation of Auditory Spatial Attention by Angry Prosody: An fMRI Auditory Dot-Probe Study

**DOI:** 10.3389/fnins.2016.00216

**Published:** 2016-05-12

**Authors:** Leonardo Ceravolo, Sascha Frühholz, Didier Grandjean

**Affiliations:** ^1^Neuroscience of Emotion and Affective Dynamics Lab, Department of Psychology, University of GenevaGeneva, Switzerland; ^2^Department of Psychology, Swiss Center for Affective Sciences, University of GenevaGeneva, Switzerland; ^3^Department of Psychology, University of ZurichZurich, Switzerland

**Keywords:** attention, spatial hearing, fMRI, voice, prosody, anger

## Abstract

Emotional stimuli have been shown to modulate attentional orienting through signals sent by subcortical brain regions that modulate visual perception at early stages of processing. Fewer studies, however, have investigated a similar effect of emotional stimuli on attentional orienting in the auditory domain together with an investigation of brain regions underlying such attentional modulation, which is the general aim of the present study. Therefore, we used an original auditory dot-probe paradigm involving simultaneously presented neutral and angry non-speech vocal utterances lateralized to either the left or the right auditory space, immediately followed by a short and lateralized single sine wave tone presented in the same (valid trial) or in the opposite space as the preceding angry voice (invalid trial). Behavioral results showed an expected facilitation effect for target detection during valid trials while functional data showed greater activation in the middle and posterior superior temporal sulci (STS) and in the medial frontal cortex for valid vs. invalid trials. The use of reaction time facilitation [absolute value of the Z-score of valid-(invalid+neutral)] as a group covariate extended enhanced activity in the amygdalae, auditory thalamus, and visual cortex. Taken together, our results suggest the involvement of a large and distributed network of regions among which the STS, thalamus, and amygdala are crucial for the decoding of angry prosody, as well as for orienting and maintaining attention within an auditory space that was previously primed by a vocal emotional event.

## Introduction

While animals such as mammals and birds use vocalizations to communicate with other conspecifics, humans tend to communicate by relying mainly on speech. For this reason, speech and language are the subject of numerous studies in psychology and cognitive neuroscience (Hickok and Poeppel, 2007), whereas prosody—particularly emotional prosody—has been less studied. The term prosody refers to the unfolding of the pitch and intensity of the human voice, as well as other specific features of voice quality (Scherer, 1986; Patel et al., 2011). In other words, prosody defines the way we say something independently of *what* we are saying (Grandjean et al., 2006; Leitman et al., 2010; Witteman et al., 2012) and it is often referred to as the melody of the human voice. The relative underrepresentation of the study of emotional prosody in the literature is rather surprising since the ability to accurately decode it in everyday life is important for human communication. In fact, prosody delivers important information, particularly about the emotional state of the sender or speaker, and we will refer to “emotional prosody” for this specific reason throughout this article. In order to understand (a) how emotional prosody influences the processing of other auditory stimuli in space (spatial orienting) and (b) to investigate how the temporal cortex is involved in such processing, we designed an auditory, diotic listening functional magnetic resonance imaging (fMRI) experiment in which we created an acoustic version of the dot-probe paradigm whereby simultaneously presented neutral and angry prosody preceded a neutral target (a sine wave tone) in the auditory space (left/right).

Human attentional systems are usually defined by two large categories across sensory modalities, namely bottom-up and top-down attention. In the case of visual and auditory modalities, bottom-up attention relates to stimulus-driven, automatic attentional capture based on exogenous cues such as saliency for instance. Top-down attention is a task-driven, voluntary attentional mechanism in which endogenous cueing orients attention, such as a specific task instruction or cue, for instance an arrow indicating the expected location-related focusing of attention. In the present study, we expected to observe enhanced brain activity in regions known for their involvement in the automatic processing of emotional prosody, namely in the superior temporal sulcus/gyrus (STS/STG) (Grandjean et al., 2005, 2008; Frühholz et al., 2012; Ceravolo et al., 2016) and the amygdala (Sander et al., 2005). This neural processing is modality-dependent and it is paralleled by activity in the fusiform face area (Kanwisher et al., 1997) and the visual cortex (Pourtois and Vuilleumier, 2006) in the visual literature in which threat was conveyed by fearful faces, even though angry faces were shown to capture attention as well (Belopolsky et al., 2011). Regarding modality-dependent top-down or voluntary attention, frontal regions have been shown to play an important role, namely the orbitofrontal cortex (OFC) in the explicit processing of emotional prosody (Sander et al., 2005) and the prefrontal lobe in the visual domain (Corbetta and Shulman, 2002). Other brain areas seem to interestingly play a role in both visual and auditory modalities and are thus defined as modality-independent. For bottom-up attention, neural commonalities for emotional content were found in the amygdala (Sander et al., 2005; Vuilleumier, 2005) while top-down attention was underlied by a converging modality-independent activity in the lateral parietal lobe such as in the posterior parietal cortex (PPC) (Corbetta and Shulman, 2002; Bisley and Goldberg, 2003; Shomstein and Yantis, 2006) and the inferior parietal lobule (IPL) (Hopfinger et al., 2000; At et al., 2011).

The common view in emotion research relies on the fact that cues related to the emotional tone of the voice are biologically relevant for humans, as are emotional facial expressions in the visual domain, because they are reliable vectors of information related to biological survival through communication mechanisms. As a consequence, emotional cues in vocal events seem to be prioritized in the processing stream (bottom-up attentional capture), especially when they imply a potential threat to the listener (see Vuilleumier, 2005, for aspects related to the concept of emotional attention). Because visual attentional tasks have only few counterparts in the auditory literature, less is known about how emotionally relevant auditory stimuli, such as angry prosody, are filtered by the attentional system or how they can influence and modulate it, and whether these relevant auditory stimuli exhibit the same enhanced processing as threatening faces do.

In fact, no imaging study to date has investigated the potential influence of spatialized angry prosody on auditory spatial attention, which is the specific aim of the present study. In particular, the underlying brain network supporting exogenous spatial attention to auditory emotional events is not yet well understood. Our task was designed to test the hypothesis according to which the detection of a neutral auditory target (a sine wave tone) would exhibit facilitated processing [e.g., faster reaction times (RTs)] when presented in a spatial location matching a previously presented angry prosody cue's spatial location. This facilitation effect would however not be true for either neutral cues or when the preceding angry prosody cue did not appear in a spatial location matching that of the following target. We also explored whether such behavioral facilitation would rely on voice-related, temporal (STS/STG) and frontal regions (OFC) related to modality-dependent bottom-up and top-down processing, respectively, as well as on modality-independent bottom-up (amygdala) and top-down attentional brain areas (PPC/IPL).

## Materials and methods

### Participants

Seventeen right-handed, healthy, native or highly proficient French-speaking participants (8 male, 9 female, mean age 24.29 years, SD 4.87) were included in this fMRI study among a sample of 19 participants, two of whom were excluded from the analyses because of below-chance performance (~25%). All included subjects had normal or corrected-to-normal vision, normal hearing, and no history of psychiatric or neurologic incidents. Participants gave written informed consent for their participation in accordance with ethical and data security guidelines of the University of Geneva. The study was approved by the Ethical Committee of the University of Geneva and conducted according to the Declaration of Helsinki.

### Stimuli

Ten professional actors (5 male and 5 female) pronounced “Aah's” (duration 1100 ms), expressing either angry or neutral prosody, providing 20 stimuli in total. These stimuli were taken from the large and validated Geneva Multimodal Expression Portrayals (GEMEP) database (Bänziger and Scherer, 2007) and were additionally evaluated by our participants (Supplementary Figures 1, 2). Stimuli were mean normalized in intensity (70 dB sound pressure level).

To present auditory voices spatialized in one auditory hemifield, we carried out a lateralization process with an average head-related transfer function (HRTF), using Panorama 5 toolbox implemented in Sony SoundForge software (Sony Creative Software Inc., Middleton, WI, USA) and parameters from the CIPIC database (Algazi et al., 2001). This convolution takes into account head and ear shape and uses wave amplitude and interaural time difference in order to virtually spatialize sounds, hence mimicking real-life auditory perception. This convolution was ideal to accurately virtually lateralize/spatialize our prosody cues as well as the target sine wave tone (SWT), meaning that even though the sound was actually in the left auditory space, it was presented to both ears with a slight delay for the ear opposite to the space of presentation (the right ear in this example). The use of an HRTF to create a diotic as opposed to a dichotic stimulus presentation significantly improved ecological validity and the procedure takes into account a double dissociation, which suggests that different neural networks serve the detection ability of the auditory space vs. that of the ears (Clarke and Thiran, 2004).

### Experimental design

**Figure 1:**
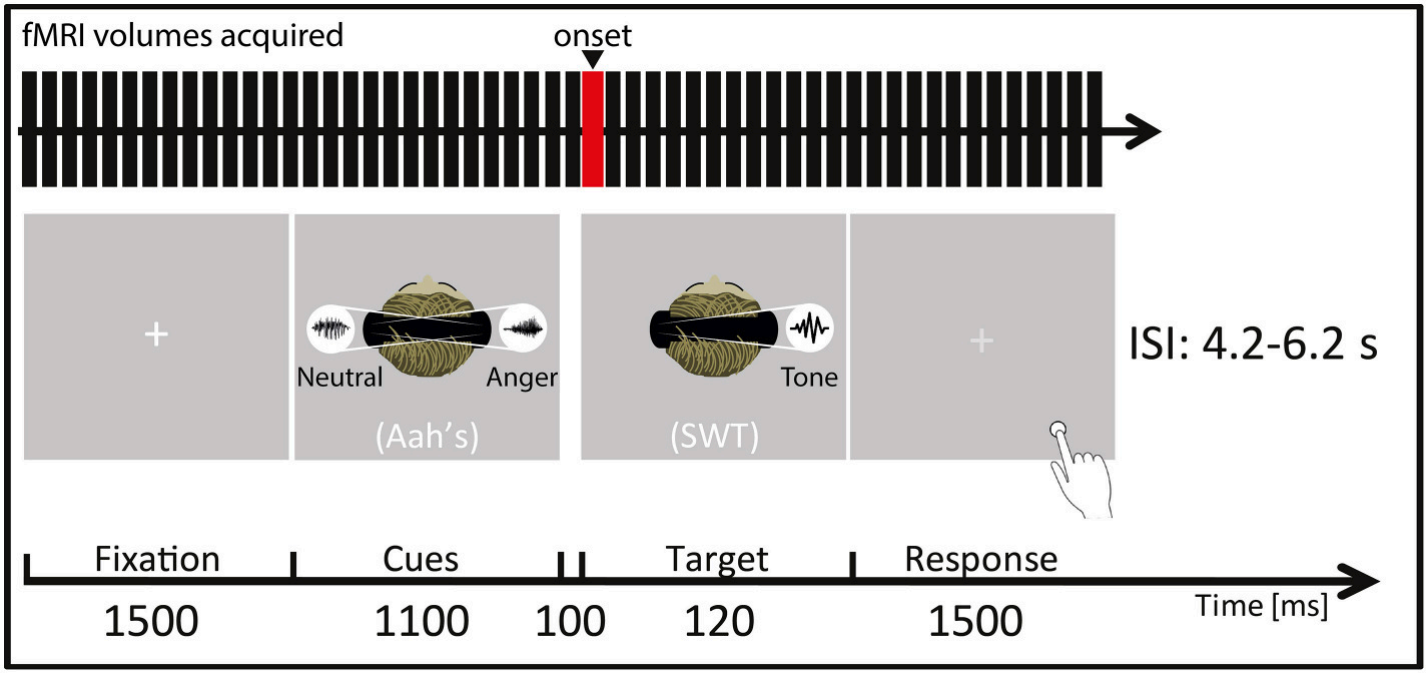
**Participants were placed in a supine position in the MRI scanner (Siemens Trio, Erlangen, Germany) and instructed to focus on the laterality of the auditorily presented target sine wave tone (SWT, 120 ms) that followed (100 ms interval) the simultaneously presented “Aah's” prosody cues (Cues, 1100 ms)**. They were instructed to focus on a white central fixation cross (Fixation, 1500 ms) displayed via a rear-mounted projector and viewed through a 12-channel head coil-mounted mirror. After the SWT offset, this white crosshair turned light gray, indicating that the participants had to give their response (Response, 1500 ms) by indicating by a key press (Response box: Current Designs Inc., Philadelphia, PA, USA) whether the target SWT appeared in the left/right auditory space. Auditory stimuli were presented through MR-compatible pneumatic headphones (MR confon GmbH, Germany) at a constant sound pressure level of 70 dB. ISI represents interstimulus interval, ranging from 4.2 to 6.2 s. The red bar of the “fMRI volumes acquired” represents the onset chosen for the present fMRI analyses, meaning that the hemodynamic response function was convolved at this point in time for each trial and condition. In this figure, a “valid” condition trial is illustrated as an example as the target SWT appears in the same spatial location as the preceding angry cue (namely in the right auditory space) while the neutral cue appears in the opposite spatial location.

Each trial started by a blank varying in duration (jittering in steps of 100 ms, mean = 2000 ms, minimum = 1000 ms and maximum = 3000 ms) directly followed by a 1500 ms fixation cross. Afterwards, the prosody cues (abovementioned “Aah's”) were presented in pairs and simultaneously to the two auditory hemispaces (left/right) through pneumatic MR-compatible headphones (MR confon GmbH, Germany) using Eprime 2 software (Psychology Software Tools, Pittsburgh, PA, USA) for 1100 ms. Thus, participants heard two voices at the same time for the cueing part of each trial (Figure 1): during stimulus presentation a right-lateralized angry cue was presented simultaneously to a left-lateralized neutral cue (and vice versa). More specifically, three pairs were possible for the cues: a neutral prosody in the left auditory space and an angry prosody in the right auditory space, the opposite, or a neutral prosody in both auditory spaces. These cues were closely followed by the to-be-attended target (50% of the total trials), namely, one SWT appearing either in the left *or* in the right auditory space. The SWT was a 120 ms, lateralized sine wave tone with a wave frequency of 600 Hz and it was presented 100 ms after the 1100 ms prosody cues. The experiment included three conditions represented by a combination of cues and target: (1) the target SWT appeared in a spatial location (left or right auditory space) matching the preceding angry prosody cue's location in space (valid trial); (2) the target SWT did not appear in the spatial location (left or right auditory space) matching the preceding angry prosody cue's location in space (invalid trial); or (3) the target SWT appeared after two neutral prosody cues were presented in both auditory spaces (neutral trial). The total number of trials for each condition (valid, invalid, and neutral) was 24, with 12 left-space and 12 right-space trials. The mean-interstimulus interval was 5200 ms (minimum = 4200 ms, maximum = 6200 ms). The order of condition presentation was pseudo-randomized so that the same condition would not appear more than 2 times consecutively. During the whole fMRI session, sound pressure level was kept constant to 70 dB.

The task for the participants was to determine as quickly and accurately as possible, for each trial, whether the target SWT appeared in the left or right auditory space, without any specific instruction regarding the preceding prosody cues (implicit prosody processing). Response was given by the participants through a key press on an MR-compatible response box (Current Designs Inc., Philadelphia, PA, USA) with button 1 meaning the target appeared in the left auditory space, button 2 meaning right auditory space targets. The mapping of the response buttons was randomized across participants.

### Behavioral data analysis

We computed a 3 × 2 repeated measure analysis of variance for accuracy data and RTs in response to the target SWT for “condition” and “space of presentation” factors and their interaction using Statistica 12 software (StatSoft Inc., Tulsa, OK, USA). Additional t-statistics were performed when interactions were significant in order to highlight the conditions that were driving the observed effects.

### Image acquisition

Structural and functional imaging data were acquired by using a 3T MRI scanner (Siemens Trio, Erlangen, Germany) equipped with a 32-channel head coil. A magnetization prepared rapid acquisition gradient echo sequence was used to acquire high-resolution (1 × 1 × 1 mm^3^) T1-weighted structural images (*TR* = 1900 ms, *TE* = 2.27 ms, *TI* = 900 ms). Functional images were acquired continuously by using a multislice echo planar imaging sequence (36 transversal slices in descending order, slice thickness 3.2 mm, *TR* = 2100 ms, *TE* = 30 ms, field of view = 205 × 205 mm^2^, 64 × 64 matrix, flip angle = 90°, bandwidth 1562 Hz/Px).

### Image analysis

Functional images were analyzed with Statistical Parametric Mapping software (SPM12, Wellcome Trust Centre for Neuroimaging, London, UK). Preprocessing steps included realignment to the first volume of the time series, normalization into the MNI space (Montreal Neurological Institute; Collins et al., 1994) by using DARTEL (Ashburner, 2007), and spatial smoothing with an isotropic Gaussian filter of 8 mm full width at half maximum (FWHM). To remove low frequency components, we used a high-pass filter with a cutoff frequency of 128 s.

We used a general linear model in which each trial was modeled by using a stick function and was convolved with the hemodynamic response function, and events were time-locked to the target SWT onset (see Figure 1). Separate regressors were created for each experimental condition and for behavioral RTs, included as a parametric modulator of no-interest on a trial-by-trial basis. An additional regressor included errors and missed trials, as well as behavioral RTs outside the bounds of an individually determined 98% confidence interval (these trials were also excluded from the behavioral data analyses). Finally, six motion parameters were included as regressors of no interest to account for movement in the data. The condition regressors were used to compute simple linear contrasts for each participant and condition (valid, invalid, neutral) and were then taken to a second-level analysis. The second-level analysis was performed with a 3 × 2 flexible factorial design with the factors “condition” and “space of presentation.” The “condition” factor (Valid trials: mean number of trials = 19; Invalid trials: mean number of trials = 18; Neutral trials: mean number of trials = 19) aimed at uncovering enhanced brain activity for valid as compared with invalid trials [Valid > Invalid trials] although several contrasts were tested [Valid > Neutral; Invalid > Neutral; Invalid > Valid; Neutral > Valid]. As our paradigm included left and right space presentation, we looked at brain differences between valid and invalid/neutral left-/right-space trials with the following contrasts: left-space Valid > Invalid; left-space Valid > Neutral; right-space Valid > Invalid; right-space Valid > Neutral. In this second-level analysis, subjects were assumed independent while it was not the case for the “condition” and “space of presentation” factors. Variance was assumed unequal for all factors (subjects, condition, space of presentation).

We were furthermore interested in the impact of the facilitation effect of valid trials on functional brain activations. Therefore, we performed a separate analysis in which we included, for each participant, the absolute value of the normalized (Z-score) mean difference between the reaction times of valid and invalid and neutral trials [valid-(invalid+neutral)] as a group covariate at the second level of analysis. In this analysis, the higher the value of the covariate, the larger the difference between valid and invalid+neutral trials was observed for the participant. Hence, this analysis allowed us to take into account the variations regarding participants' individual facilitation effects, where this effect could be stronger or weaker for each participant. In order to get statistically correct results, this analysis was conducted using contrasts between conditions at the first level (Valid>Invalid or [1 −1 0]). For each participant, this contrast was then taken to a one-sample *t*-test second-level analysis in addition to having the facilitation covariate defined in the model. We could then display the Valid > Invalid contrast by taking into account the impact of the behavioral facilitation as a covariate in the GLM.

All activations are reported at a threshold of *p* < 0.005 (uncorrected) and a cluster extent threshold of k > 86 voxels, equivalent to a Family-Wise Error correction for multiple comparison of *p* < 0.05 at the cluster level. This threshold was based on the final FWHM of the data (11.3, 11.3, 10.7 mm), using the 3dClustSim function in AFNI software (Cox, 1996; http://afni.nimh.nih.gov/afni), using a non-parametric method with 10,000 iterations to estimate the necessary cluster extent thresholding for side-to-side voxels (NN-2 option). 3dClustSim reports a cluster extent threshold for each specified statistical *p*-value and follows the assumption that neighboring voxels are part of a similar functional response pattern, rather than a completely different and independent measure as implied by the family-wise error correction at the single voxel level.

Functional statistical images are displayed on the “152 average T1” mean anatomical image as part of SPM12 sections, and brain-non-brain tissue separation was performed using Extract Brain (BET) plugin in Mango software (http://ric.uthscsa.edu/mango/mango.html, Research Imaging Institute, UTHSCSA). Anatomical locations were defined with a standardized coordinate database (Talairach Client, http://www.talairach.org/client.html) by transforming MNI coordinates to match the Talairach space and transforming it back into MNI for display and precision purposes.

## Results

### Behavioral data

Accuracy data for each condition (valid, invalid, neutral) showed no performance differences [*F*_(2, 32)_ = 0.97, *p* = 0.39], whereas there was a difference for the space of presentation factor [*F*_(1, 16)_ = 7.10, *p* = 0.017], showing significantly higher accuracy for left-space than for right-space trials [*t*_(16)_ = 2.66, *p* = 0.017]. The interaction between condition and space of presentation was not significant [*F*_(2, 32)_ = 0.16, *p* = 0.85] (see Table 1).

**Table 1:** **Reaction times of correct responses and accuracy data for all participants (***N*** = 17) and each condition and space of presentation**.

**Condition**	**Reaction times in ms (*SD*)**	**Percentage (*SD*)**
Valid left Valid right	462 (160) 477 (135)	84 (17) 71 (15)
Invalid left Invalid right	484 (158) 529 (218)	82 (15) 67 (19)
Neutral left Neutral right	485 (185) 523 (196)	84 (18) 68 (21)

**Figure 2:**
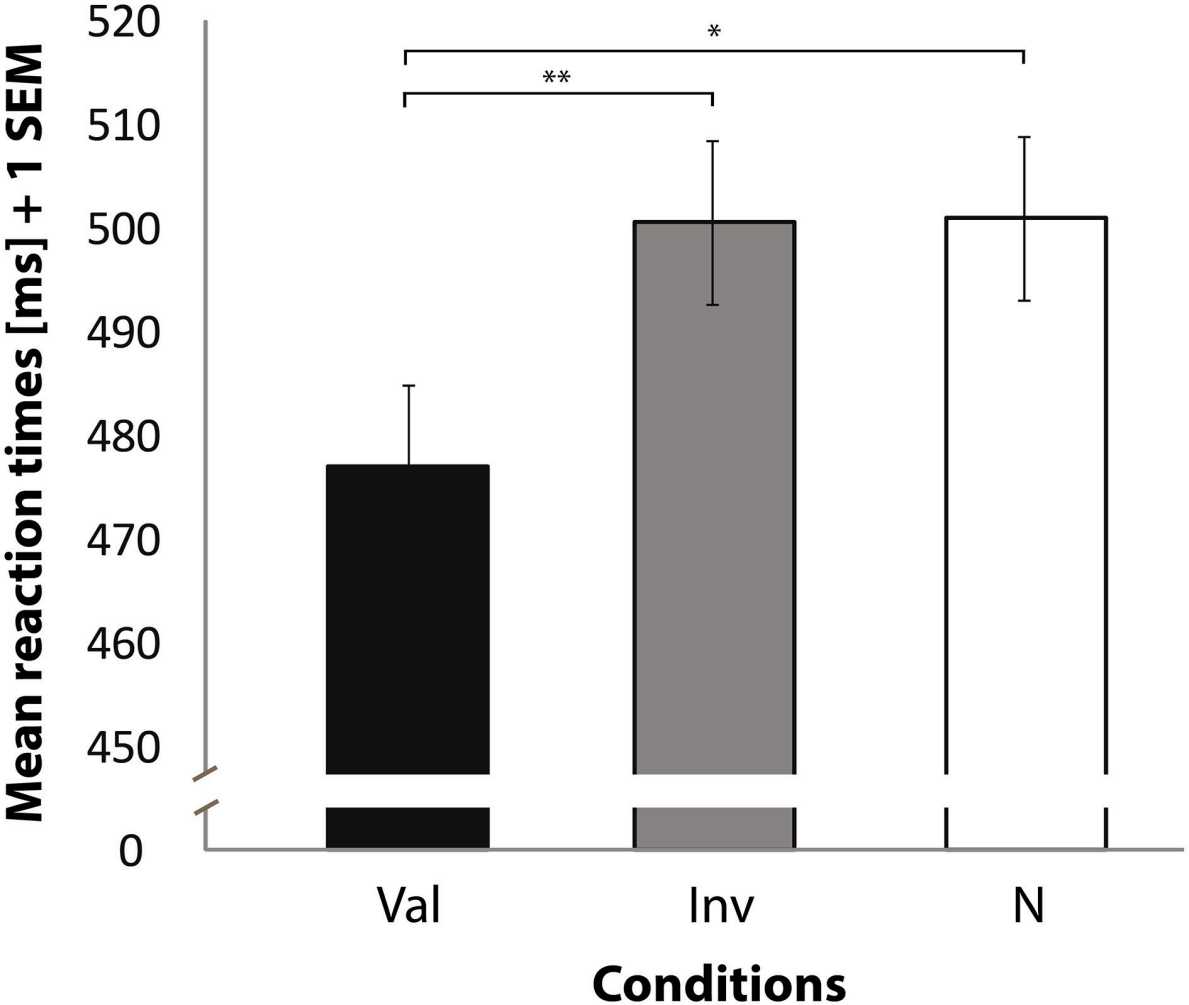
**Mean reaction times (Y axis) for correctly locating the target sine wave tone**. Bars represent valid (Val), invalid (Inv), and neutral (N) conditions (X axis). Data used for these analyses are within the bounds of a 98% confidence interval. Error bars indicate the standard error of the mean. ^*^p < 0.05. ^**^p < 0.01.

RTs of correct trials to localize the SWT revealed differences between conditions [*F*_(2, 32)_ = 4.89, *p* = 0.014] (see Figure 2), and left-space trials had faster RTs than right-space trials [*F*_(1, 16)_ = 7.86, *p* = 0.013]. No significant interaction was observed between condition and space of presentation [*F*_(2, 32)_ = 0.89, *p* = 0.42]. As we were interested in a validity effect, we performed paired *t*-tests on the basis of the abovementioned significant condition differences. This analysis revealed faster RTs when valid trials were compared with invalid [*t*_(16)_ = −2.96, *p* = 0.009] and neutral trials [*t*_(16)_ = −2.18, *p* = 0.044]. No significant difference was found when invalid trials were compared with neutral trials [*t*_(16)_ = 0.22, *p* = 0.83]. A comparison of valid against invalid and neutral trials also revealed a significant difference [*t*_(16)_ = −2.70, *p* = 0.016]. In addition, a significant difference was found when invalid trials were compared against valid and neutral trials [*t*_(16)_ = 2.29, *p* = 0.036]. Finally, no difference was found for neutral compared to the average of valid and invalid trials [*t*_(16)_ = 1.33, *p* = 0.201].

### Whole-brain functional data

#### Neuroimaging results of spatially matching/non-matching prosody cues and tone target

**Figure 3:**
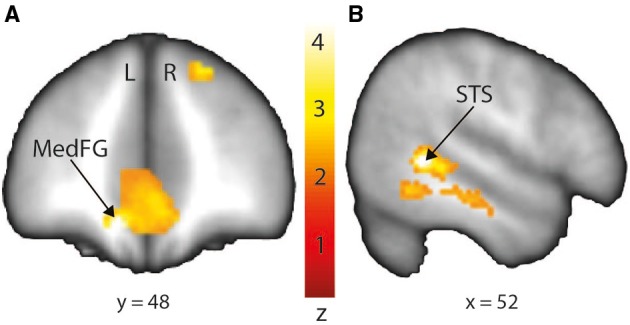
**Stronger activation to valid compared with invalid trials in the medial frontal gyrus (MedFG) and in the right posterior superior temporal sulcus (STS) shown on a coronal (A) and sagittal (B) slice, respectively**. Posterior STS: MNI *x* = 52; *y* = −46; *z* = 2. MedFG: MNI *x* = −10; *y* = 48; *z* = −12. The colored bar shows the normalized value of activation (Z-score).

In order to interpret our behavioral results in terms of specific brain regions underlying an attentional facilitation effect, we relied on specific contrasts, notably valid compared with invalid trials and valid compared with invalid trials when taking into account the space of presentation of the stimuli. The first contrast was used to emphasize an effect of auditory spatial attention or spatial orienting, as both valid and invalid trials contain the same emotional content, although cuing was crucially different (spatial matching between cue and target for valid trials; absence of such spatial matching for invalid trials). A comparison of valid and invalid trials showed increased activation in bilateral STS, with the highest activation in the right posterior STS (Figure 3B; Table 2), as well as an enhanced BOLD signal in the medial frontal (MedFG) and superior frontal gyri (Figure 3A). Interestingly, STS regions showed an enhanced BOLD signal for valid compared with invalid trials, while a smaller decrease of activation was observed in the MedFG for valid compared with invalid trials. No above-threshold activity was observed when the inverse contrast was computed (invalid > valid) or when invalid trials were compared to neutral trials (invalid > neutral). For valid compared to neutral trials, one cluster in the right posterior superior temporal sulcus showed enhanced activity (Supplementary Figure 3). This cluster interestingly overlaps with the pSTS region found in the Valid > Invalid contrast.

**Table 2:** **Mean cluster location and local maxima of BOLD signal change for valid compared with invalid trials**.

**Region name (Brodmann area)**	**Left/Right (L/R)**	**Z-score**	***X***	***Y***	***Z***	**Size (voxels)**
Superior temporal sulcus (22)	R	4.42	52	−46	2	905
Inferior occipital gyrus (18)	R	4.40	32	−90	−12	428
Medial frontal gyrus (11)	R	4.06	4	32	−12	839
Parahippocampal gyrus (28)	L	3.81	−22	−18	−22	160
Superior frontal gyrus (8)	R	3.91	20	42	42	505
Posterior cingulate (29)	R	3.54	8	−42	20	592
Superior temporal gyrus (22)	L	3.51	−44	−24	−4	198
Middle frontal gyrus (8)	L	3.28	−48	16	44	143
Superior frontal gyrus (6)	L	3.11	−18	20	60	109
Fusiform gyrus (37)	L	3.10	−50	−46	−16	140

**Figure 4:**
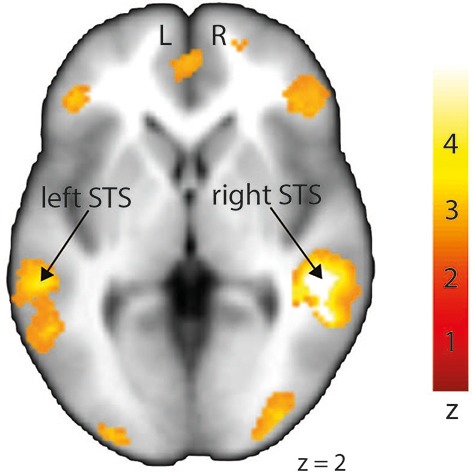
**Stronger activation to right-space valid trials compared with right-space invalid trials in the left and right middle superior temporal sulcus (STS) shown on a transverse slice**. Left STS: MNI *x* = −60; *y* = −36; *z* = −2. Right STS: MNI *x* = 42; *y* = −40; *z* = 2. The colored bar shows the normalized value of activation (Z-score).

In order to investigate differences in brain activity regarding the space of presentation of the validly cued trials, we computed contrasts for left-space and right-space trials. Taking into account the space of presentation did not yield any above-threshold voxels when we contrasted left-space valid trials with left-space invalid or neutral trials or when comparing invalid with neutral trials (left-space > left-space *or* right-space > right space trials). However, computing the valid > invalid contrast with right-space trials revealed broad activity in the bilateral STS, the MedFG, the inferior frontal gyrus (IFG) (Figure 4), and the IPL (see Table 3 for details).

**Table 3:** **Mean cluster location and local maxima of BOLD signal change for right-space valid compared with right-space invalid trials**.

**Region name (Brodmann area)**	**Left/right (L/R)**	**Z-score**	***X***	***Y***	***Z***	**Size (voxels)**
Superior temporal sulcus (22)	R	4.49	52	−44	2	1799
Middle temporal gyrus (21)	R	4.04	64	−12	−14	
Superior frontal gyrus (8)	R	4.52	20	40	44	2186
Middle frontal gyrus (47)	R	4.20	42	36	−6	1686
Inferior frontal gyrus (46)	R	3.50	50	28	12	
Middle frontal gyrus (11)	L	4.15	−30	40	−10	921
Inferior frontal gyrus (45)	L	2.97	−50	28	10	
Superior temporal sulcus (21)	L	3.92	−60	−36	−2	1008
Middle temporal gyrus (21)	L	3.27	−60	−20	−8	
Inferior occipital gyrus (18)	R	3.83	42	−84	−8	341
Inferior parietal lobule (40)	R	3.74	54	−44	52	394
Posterior cingulate gyrus (31)		3.59	0	−30	34	643
Inferior occipital gyrus (18)	L	3.36	−30	−92	−4	300
Supramarginal gyrus (40)	L	3.27	−62	−50	34	456
Precuneus (7)	L	3.25	−4	−74	36	100

#### Neuroimaging results of spatially matching/non-matching prosody cues and tone target as a function of the magnitude of the participant-specific behavioral facilitation effect

Abovementioned results looked at the contrasts between conditions without taking into account participant-specific behavioral facilitation as a group covariate. Thus, behavioral facilitation was added here as a group covariate at the second level of analysis in order to explore brain activity linearly related to the magnitude of the individual facilitation effect for each participant.

**Figure 5:**
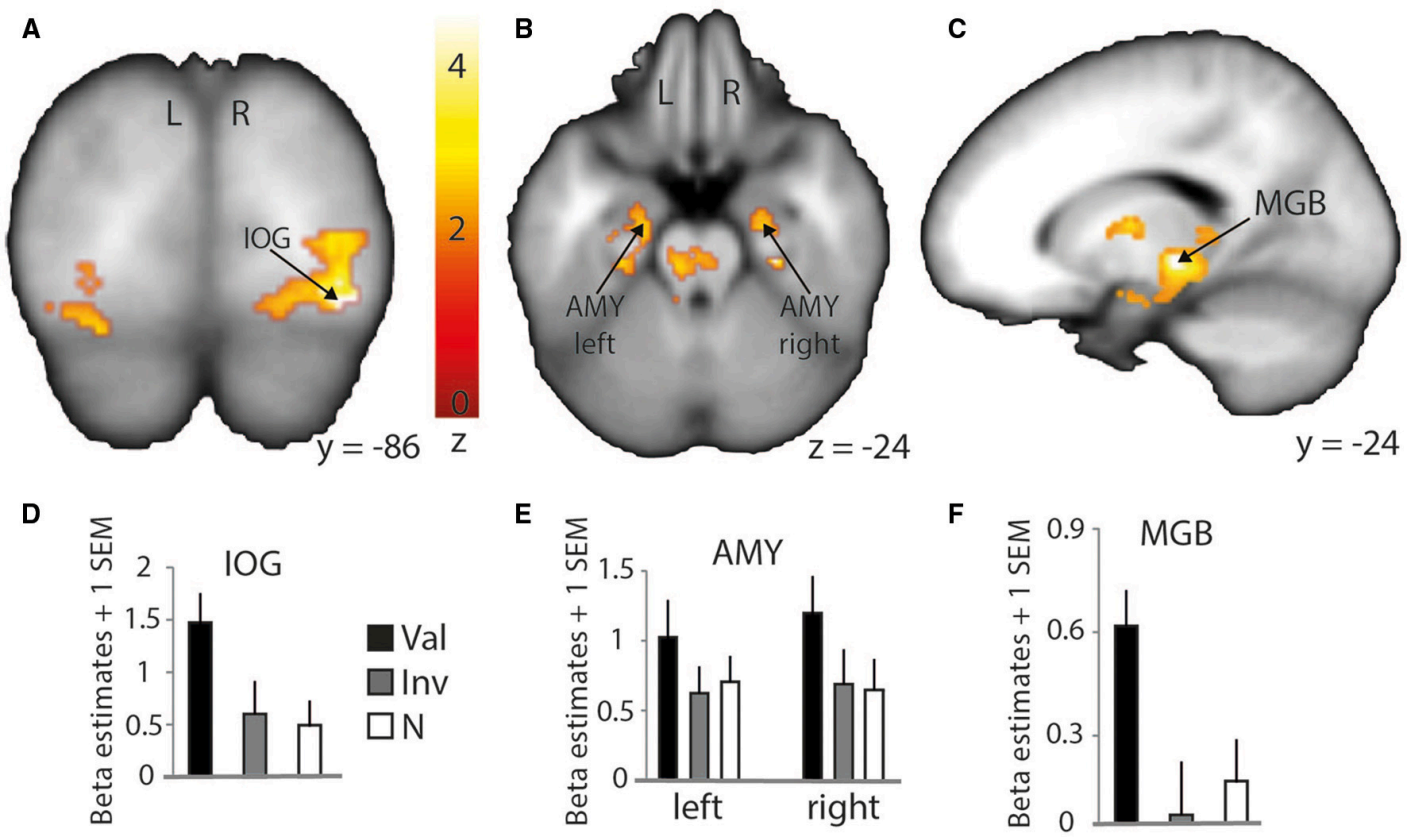
**Stronger activation to valid compared with invalid trials with behavioral (reaction time) facilitation as a group-level parametric modulator**. Increased BOLD signal in the right inferior occipital cortex (IOG) is shown on a coronal slice **(A)** and in the amygdalae (AMY) and medial geniculate body (MGB) on a transverse **(B)** and sagittal slice **(C)**, respectively. Percentage of signal change in the right IOG **(D)** (MNI *x* = 34; *y* = −86; *z* = −12), left and right amygdalae **(E)** (left: MNI *x* = −22; *y* = −13; *z* = −24; right: MNI *x* = 22; *y* = −12; *z* = −24), and left thalamus/medial geniculate body **(F)** (MNI *x* = −16; *y* = −24; *z* = −4). Bars represent valid (Val), invalid (Inv), and neutral (N) conditions. Error bars represent the standard error of the mean (SEM). The colored bar shows the normalized value of activation (Z-score).

Although contrasting valid against invalid trials revealed cortical activations that were similar to the previously presented results (Table 4; medial frontal and temporal cortex), it greatly extended the impact of visual and subcortical regions on attentional processes involved in validly cued vs. invalidly cued trials (Figure 5). An enhanced BOLD signal was observed in the thalamus, more specifically in the left medial geniculate body (MGB) (Figure 5C), bilaterally in the posterior part of the amygdala (Figure 5B), and in the right inferior occipital cortex (IOG) (Figure 5A). Additional activations were found in the caudate tail and parahippocampal gyri (see Table 4 for details). While these results partly overlap with those of Section Neuroimaging Results of Spatially Matching/Non-Matching Prosody Cues and Tone Target, they emphasize the crucial impact of inter-individual orienting facilitation effects on the neural activity of validly cued target detection accuracy.

**Table 4:** **Mean cluster location and local maxima of BOLD signal change for valid compared with invalid trials with reaction time facilitation effect as a group-level covariate**.

**Region name (Brodmann area)**	**Left/Right (L/R)**	**Z-score**	***X***	***Y***	***Z***	**Size (voxels)**
Inferior temporal gyrus (37)	L	4.28	−42	−50	−12	275
Inferior occipital gyrus (1)	R	4.12	32	−90	−10	312
Posterior cingulate gyrus (29)	R	4.08	10	−44	22	757
Superior temporal suclus (2)	L	4.05	−44	−24	−6	376
Parahippocampal gyrus (36)	L	4.05	−24	−26	−22	258
Amygdala			−22	−13	−24	
Medial frontal cortex (11)	L	4.02	−12	48	−14	612
Parahippocampal Gyrus (36)	R	3.91	24	−20	−12	207
Amygdala			22	−12	−24	
Caudate tail	R	3.55	34	−16	6	131
Lingual gyrus (18)	L	3.41	−28	−96	−4	108
Medial Geniculate body ()	L	3.39	−16	−24	−4	225

## Discussion

In the present study, we investigated how the presentation of spatialized angry prosody could influence subsequent target detection (i.e., a sine wave tone) in the auditory space. We used a variant of an original auditory dot-probe paradigm. Here, participants simultaneously heard lateralized voice utterances in both ears, including angry or neutral voices, followed by a lateralized tone that they had to localize in the left or right auditory space. The main goal of the study was to reveal the behavioral and neural influence of angry prosody on auditory spatial attention by an exogenous cueing followed by target detection in an ecologically-valid diotic listening task. A facilitation effect was observed specifically for spatially matching angry cue-target tone occurrences (valid trials), while fMRI data highlighted the role of the middle and posterior parts of the STS/STG and medial frontal gyrus (MedFG) for auditory spatial attention orienting in valid vs. invalid and valid vs. neutral trials. A second type of analysis with the facilitation effect as a group-level covariate revealed an enhanced BOLD signal in additional regions such as the IOG, the amygdalae, the MGB of the thalamus, the lentiform nucleus, and the caudate tail. Our results reveal a broad network of brain regions underlying attentional mechanisms of spatial orienting triggered by angry voice presentation and emphasize the impact of individual behavioral facilitation as a group-level participant specification.

### Behavioral data

Our behavioral data showed better-than-chance performance for all conditions and no significant difference between them in terms of accuracy, while left-space trials had higher accuracy than right-space trials. Significantly faster RTs were observed for valid as compared with invalid and neutral trials, while no significant difference was observed between the RTs of invalid and neutral trials. RTs for invalid trials were also slower than RTs of valid and neutral trials taken together. Consequently, an important aspect of the present study relies in trial conception. In fact, both valid and invalid trials contained the same type of angry and neutral prosodies preceding the target tone, but the behavioral facilitation effect was nevertheless observed exclusively when the cue and the target were presented in a matching auditory space, and when the spatially matching cue was an angry prosody (valid trials). In addition, there was no possible prediction regarding the occurrence and the space of presentation of a target following the prosody cues, as no endogenous cueing was used and the target appeared in only 50% of the trials. This feature reflects an important difference compared with dichotic listening paradigms, which usually include a to-be-attended vs. an unattended ear for processing stimuli (Grandjean et al., 2005; Brosch et al., 2008), thus priming and influencing top-down control of attention.

In other words, our behavioral data show a facilitation effect for valid trials only. This might be explained by the fact that individuals reflexively extract the relevant and salient information in angry voices. This information serves as an exogenous cue to further direct and maintain spatial attention within a specific auditory hemispace, leading to faster detection of an auditory target if it appears in the same hemispace as a preceding angry voice. Thus, spatial orienting could be triggered by the emotional tone of the vocal cue, by some low level acoustic cues, or more probably it could be due to the concomitant apparition of a particularly significant event in space, in the present case an angry voice. The observed facilitation effect for valid trials is comparable to similar data reported in the visual domain (Pourtois and Vuilleumier, 2006) and it is also in line with a cross-modal dot-probe study involving emotional prosody as a cue to a visual target (Brosch et al., 2008). Thus, these results validate our choice of using angry prosody as a spatial vocal cue. Moreover, angry voices were reported as more accurately recognized among other threatening emotions such as fear (Banse and Scherer, 1996).

In the present study, we have demonstrated that such a facilitation effect is also possible in a unimodal auditory paradigm, and these data extend previous results and draw interesting parallels with research on spatial attention in the visual domain. More work should however be conducted to shed light on the potential automatic processing of threat-related stimuli, independent of attention, as our results can only partly respond to this issue with the use of a single emotion. Furthermore, our conditions confounded attentional and emotional effects. While this manipulation was voluntary in our study, double cueing methods or a fine-tuned paradigm that differentiates between these factors would improve the understanding of the modulation of spatial attention by emotionally tinted content. Finally, regarding neuroimaging acquisition, the use of continuous (the MRI scanner does not stop while a stimulus is presented) over sparse (the MRI scanner stops while a stimulus is presented) sampling acquisition can be discussed as well, as scanner noise can of course impact auditory perception. While an ideal neuroimaging data acquisition would have been sparse sampling, in our study all conditions were presented while the scanner was running. The main reason for deciding to use continuous as opposed to sparse sampling scanning was related to the cue, as we wanted to leave the possibility of analyzing not only neuroimaging data of the target (the tone) but also related to the cue (the voice prosodies), open. While scanner noise could hence have biased the perception of lateralized stimuli, another potential explanation for this difference regarding accuracy between left-space compared to right-space trials would involve HRTF convolution. In fact, we used an ecologically-valid convolution of our auditory signals in order to virtually spatialize them, but this convolution was not specific to each participant and was an average (of yet an independent group; see Algazi et al., 2001 for details). Hence, because HRTFs were not individually-matched, it is highly possible that perception was somehow varying across participants and within the space of presentation factor, leading to a difference in accuracy for left-/right-space trials. The direction of this difference can, however, not be interpreted as accuracy could have been higher for right- as opposed to left-space trials. In a recent study (Ceravolo et al., 2016), we indeed obtained extremely similar behavioral results using a voice-distance evaluation task in both an MRI group (using continuous scanning) and an independent control group using semi-individualized HRTFs. Hence, despite scanner noise, task accuracy was nevertheless significantly above chance level and our interpretation is that semi-individualized HRTFs helped accurate auditory perception, be it in the scanner or in a control experimental room.

### Brain regions underlying implicit angry prosody processing and explicit target detection

The present auditory dot-probe task involved both the implicit processing of voice prosody and the explicit processing of a target sound. We expected increased activation in areas known to be involved in processing tones and especially voices, namely, the higher level auditory cortex, an associative region known to be highly sensitive to human voices (Belin et al., 2000) and to emotionally angry prosody (Grandjean et al., 2005; Frühholz et al., 2012). We interpret the behavioral facilitation effect as being triggered by the matching between auditory space and angry voices/target, as discussed earlier. Following this reasoning, the STS/STG regions activated by angry voices would be biased or primed to preferentially process the specific auditory space where the angry voice cue appeared, even though a neutral prosody cue was also presented in the opposite auditory hemispace.

When contrasting valid with invalid trials, we indeed found increased activation in bilateral middle and posterior parts of the STS (pSTS), as well as decreased deactivation in distinct subregions of the medial part of the frontal cortex. The pSTS was also involved in the valid > neutral comparison, strengthening the role of this region in processing spatially congruent auditory information. Interestingly, the most significant peak voxels of this analysis revealed activations in STS/STG regions that were located within the voice-sensitive areas (Belin et al., 2000). This finding suggests a broader role for these voice regions rather than the perception and processing of acoustical features of voices and speech prosody (Ethofer et al., 2006). Our results fit well with other studies on emotional prosody processing showing that mid STS regions were sensitive to emotional voices, independently of endogenous spatial attention (Grandjean et al., 2005; Sander et al., 2005; Frühholz et al., 2012; Witteman et al., 2012; Ceravolo et al., 2016).

Moreover, our results also took the offside of presentation into account even though the aim of the study was a general effect of facilitation through cue/target involving space-related validity. While left-space valid trials did not show any difference from left-space invalid trials, right-space trials did show differences. Indeed, right-space valid against invalid trials showed enhanced activation in brain regions that were similar to those obtained with the contrast within the condition factor (valid > invalid). This result emphasizes the role of right-space trials in our neuroimaging data despite their lower accuracy and slower RTs compared with left-space trials. This result seems to denote an opposition between behavioral and imaging data that could be due to statistical thresholding for instance. In fact, neuroimaging and behavioral results were not processed using the same software (SPM12 vs. Statistica). Furthermore, studies on angry prosody perception already showed an advantage for the right hemisphere (Grandjean et al., 2005) that seems present in our data but opposes at the same time to our neuroimaging data. The present results cannot address the reasons of this incongruence and more work on auditory spatial perception is needed to clarify this matter. Finally, no subsequent lateralization of brain responses was observed, as bilateral activations were obtained for this contrast in both left and right STS regions.

The results of the present analysis including first level RTs as covariate of no interest offer new insights into the role of different subregions of the STS/STG, showing that posterior and middle regions are specifically modulated by the emotion value and may facilitate the processing of subsequent auditory stimuli presented in the same auditory hemispace. These results improve our understanding of the interaction between emotion and attention in the auditory space and the vocal domain by the use of a diotic listening task, allowing its comparison with dichotic studies that concern ears rather space (Grandjean et al., 2005; Sander et al., 2005).

### Brain regions responsible for the individual-related impact of behavioral facilitation by angry prosody

Following the previous analysis, we wanted to investigate more directly the impact of facilitation effects obtained at the behavioral level on our brain imaging results. Thus, we used the individual behavioral facilitation effect for valid trials as a covariate in our group-level analyses [absolute value of the Z-score of RT difference corresponding to valid-(invalid+neutral)] in order to observe enhanced BOLD signal specific to our conditions but also linearly varying with the magnitude of participant-specific behavioral facilitation effects. We found this procedure greatly influenced the aforementioned results, mostly by additionally involving several subcortical and visual regions.

In fact, while a clear overlap with previously discussed STG/STS regions was observed, this specific type of analysis emphasized an involvement of the bilateral amygdalae for valid trials. The role of the amygdala in visual attention paradigms has already been demonstrated in recent studies (Vuilleumier et al., 2001, 2004; Peck and Salzman, 2014) and identified as being important for the processing of angry voices (Sander et al., 2005; Frühholz et al., 2012, 2014, 2015). In the present study, it indicates a clear role of the bilateral amygdalae in facilitating the auditory space-matching detection of a target following exogenous angry prosody cuing. This result should, however, be investigated in more detail to better understand the feed-forward role of the amygdala with other brain regions. In fact, the amygdala was suggested to be a region able to modulate the activity of the visual cortex through feed-forward connections (Vuilleumier et al., 2004) and such direct link also exists in the auditory domain (Frühholz et al., 2015).

The caudate tail also showed enhanced activity for valid compared with invalid trials. The caudate nucleus has a potentially important role in perceiving emotional prosody, but previous findings highlighted the involvement of the head of the caudate rather than the tail (Kotz et al., 2003). Moreover, as shown in a recent study with cats, specific neuron populations of the caudate nucleus accurately code locations of visual stimuli (Gombkötö et al., 2011) and such coding could be paralleled in humans by the caudate tail for coding auditory locations. This explanation is however speculative at this point and further work should aim at defining the different auditory-specific attentional and perceptual functions underlied by distinct subregions of the caudate nucleus, which is a rather large subcortical brain region.

Finally, the results of this analysis with the behavioral facilitation effect used as a group covariate yielded increased activity in the left thalamus, more specifically in the medial geniculate body (MGB) as part of the ascending auditory pathway. In the guinea pig, results showed a direct pathway from the cochlear nucleus to the ventral MGB (Anderson et al., 2006). This pathway was involved in perceiving short latency clics, and its direct connection with the amygdala would more efficiently lead to a flight response in a threatening context. A similar direct pathway was observed in the rat (Malmierca et al., 2002) and further work showed that the impact of a disconnection between the MGB and the amygdala (lesions to either the MGB and/or the amygdala) has a crucial effect on associative emotional conditioning (Iwata et al., 1986). Moreover, the connection between the MGB and the amygdala was hypothesized to occur in humans in voice and music processing (Frühholz et al., 2014). The MGB seems hence to have direct efferent connections to the lateral part of the amygdala and receives afferent connections from it via the inferior colliculus, in addition to having efferent connections with the auditory cortex and the hippocampus for voice perception (Frühholz et al., 2014).

Taken together, these results suggest a widespread brain network underlying spatial attention capture and orienting that is influenced by angry voice cueing. According to our results, STS/STG regions are recruited for the automatic perception and processing of angry prosody, together with a potential allocation of cognitive resources to the auditory space through the basal ganglia (i.e., the caudate tail) and more probably through the amygdala. The ascending auditory pathway recruits the MGB that has feed-forward connections to the STS/STG. The MGB is also bi-directionally connected with the amygdala, a key region that can directly modulate the activity of the associative auditory cortex (as well as indirectly through the primary auditory cortex). In our study, this modulation is thought to take place because of the appearance of angry prosody in one auditory space, and an orienting of spatial attention to the location of angry voices would then happen, allowing faster target detection in the case of a spatial matching of cue and target events. Eventually, enhanced activity in these brain areas is triggered by taking into account the variance explained by individually-classified behavioral facilitation effects in the analysis of neuroimaging data, pointing toward an important role of inter-individual differences in spatial attentional mechanisms related to vocal, emotional events.

## Conclusion

The results of the present study emphasize the role of angry prosody in capturing attention when presented in the auditory space. The subsequent detection of a to-be-attended target is indeed facilitated when its position in space matches that of the previously presented angry prosody cue (valid trials), showing faster detection as compared to when position in space does not match the angry cue (invalid trials) or when the cue includes neutral prosody only. The present results highlight one way of orienting auditory spatial attention by angry voice cueing, but other positive/negative emotions should be studied in such context. Neuroimaging data point toward a complex and distributed network of regions underlying such behavioral orienting mechanisms, including both cortical and subcortical brain regions. Our results specifically highlight the involvement of the medial geniculate body and the amygdala, together with the superior and middle temporal regions and the medial frontal cortex, in the accurate and effective modulation of auditory spatial attention through angry prosody cueing, as illustrated by a facilitation effect (faster reaction times) at the behavioral level. This neural network still requires further investigation, notably by using functional and effective connectivity methods in auditory spatial attention paradigms in order to more specifically understand the precise functional communication between each of these distributed brain regions.

## Author contributions

LC and DG designed the study. LC and SF collected the data. LC analyzed the data. All authors interpreted the data. LC, DG, and SF wrote the manuscript.

### Conflict of interest statement

The authors declare that the research was conducted in the absence of any commercial or financial relationships that could be construed as a potential conflict of interest. The reviewer DW and the handling Editor declared their shared affiliation, and the handling Editor states that the process nevertheless met the standards of a fair and objective review.
