# Supplementary material for: Modulation of Auditory Spatial Attention by Angry Prosody: An fMRI Auditory Dot-Probe Study
Source: Front Neurosci. 2016 May 12;10:216. doi: 10.3389/fnins.2016.00216 (PMC4864064; doi:10.3389/fnins.2016.00216)
Supplement: Supplementary file 1 [file DataSheet1.PDF]

Supplementary Material

Modulation of auditory spatial attention by angry prosody: an fMRI auditory dot-probe study

Leonardo Ceravolo[*], Sascha Frühholz and Didier Grandjean

***Corresponding author:** Leonardo Ceravolo, Leonardo.Ceravolo@unige.ch

Supplementary Figures

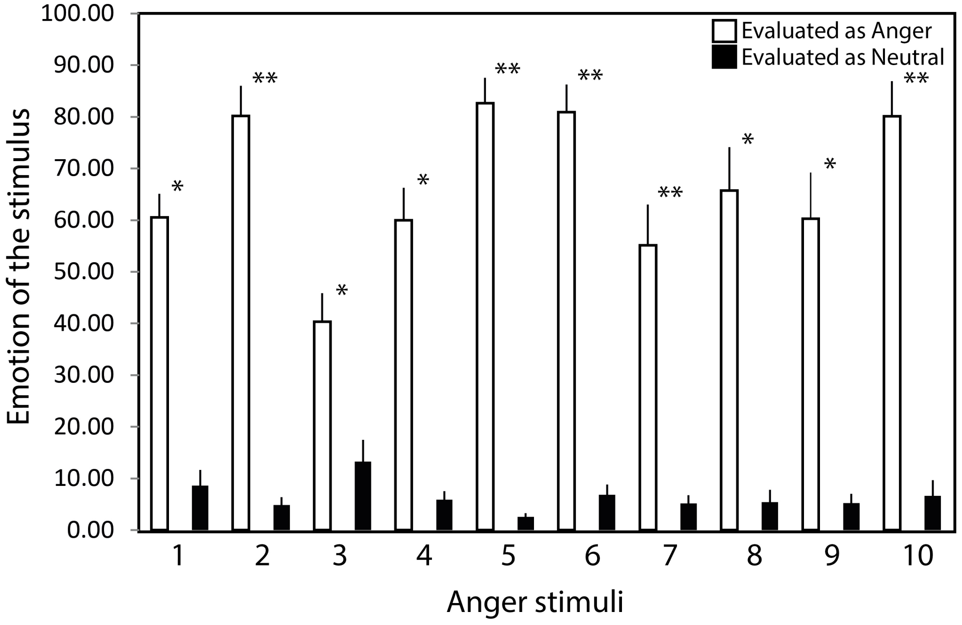

Supplementary Figure 1. Mean (N = 17) subjective evaluation of the anger/neutral content (*Y* axis) of the 10 anger stimuli used to create the exogenous prosody cues of the task (*X* axis). The evaluation was performed by moving two visual sliders, each ranging from 0 to 100, for both anger and neutral emotion potentially expressed by the presented anger stimuli. White bars represent anger stimuli being evaluated as expressing anger; black bars represent anger stimuli being evaluated as expressing neutral emotion. Error bars indicate standard error of the mean. * $p < .05$; ** $p < .01$.

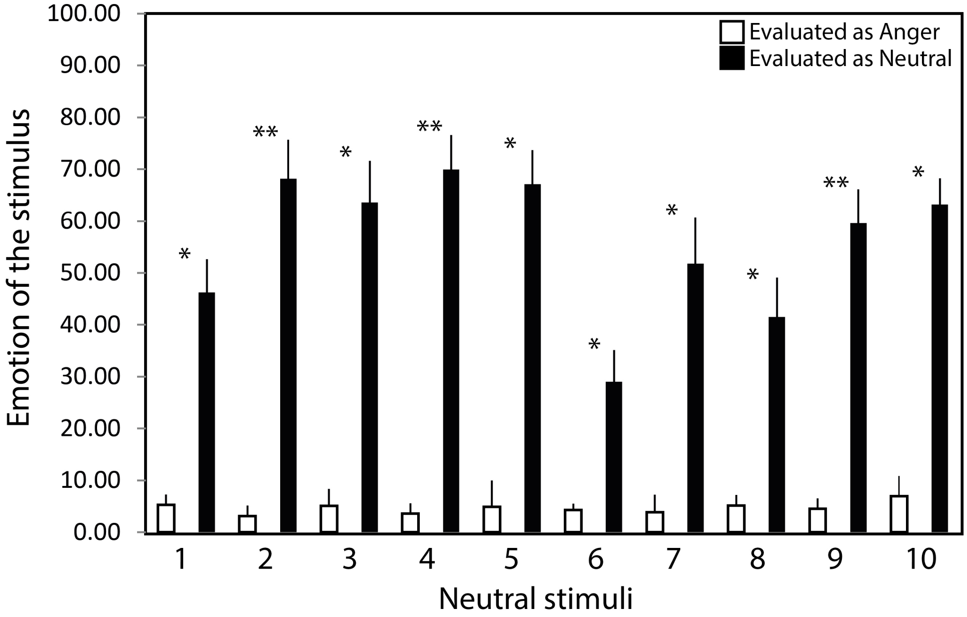

Supplementary Figure 2. Mean (N = 17) subjective evaluation of the anger/neutral content (*Y* axis) of the 10 neutral stimuli used to create the exogenous prosody cues of the task (*X* axis). The evaluation was performed by moving two visual sliders, each ranging from 0 to 100, for both anger and neutral emotion potentially expressed by the presented neutral stimuli. White bars represent neutral stimuli being evaluated as expressing anger; black bars represent neutral stimuli being evaluated as expressing neutral emotion. Error bars indicate standard error of the mean. * $p < .05$; ** $p < .01$.

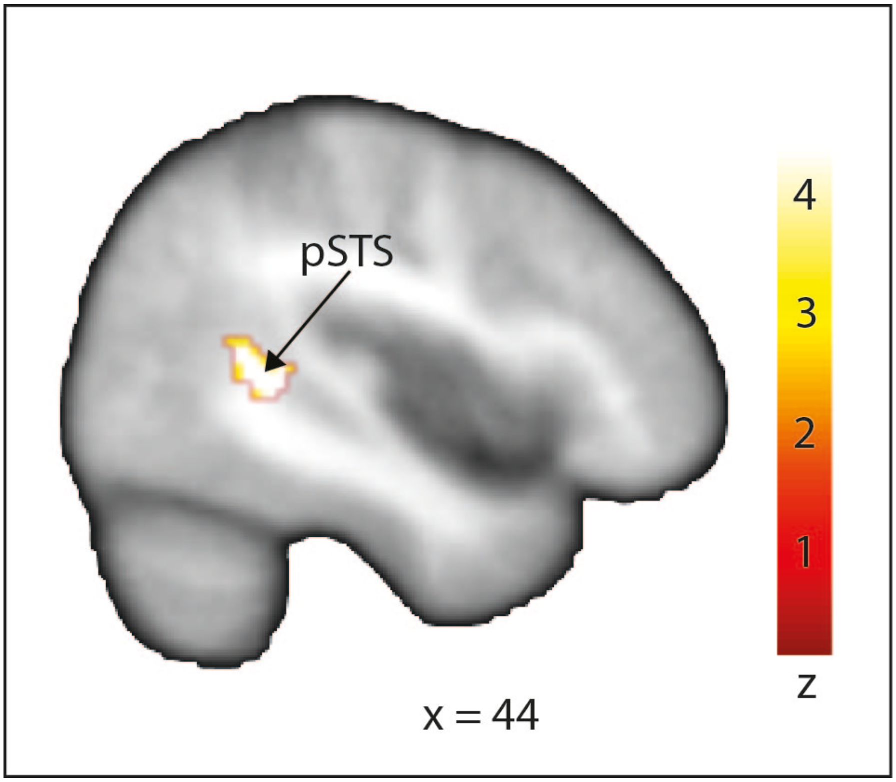

Supplementary Figure 3. Stronger activation to valid compared with neutral trials in the posterior superior temporal sulcus (pSTS) shown on a sagittal slice. Posterior STS: MNI x = 42; y = -44; z = 4. The colored bar shows the normalized value of activation (Z score).
